# The Texas Medical College—Third Session

**Published:** 1893-10

**Authors:** 


### Opening of Third Annual Session.

The Medical Department of the State University opened October 2nd, inst., Rev. Mr. Carter, of Galveston, invoking divine aid to the labors of the session. The Dean then introduced Prof. William Keiller, of the Medical Faculty, who addressed the assembled classes, welcoming them, and directing his words especially to advise proper methods of study, and to place before the students their just relations to the profession and the faculty of the school. In conclusion he placed before them the picture of the country practitioner, broad-minded, hard-working, poorly recompensed, honorable, honest, and a gentleman—the true ideal of a worthy student. The address was warmly received by the audience, and the lecturer fairly added another to his wreath of laurels.

In behalf of the Faculty of Pharmacy, Professor James Kennedy made a brief address, indicating the purposes of the new school of pharmacy and its plan of instruction, and taking occasion to express his appreciation of the State Pharmacentical Association for the part it has taken in the organization of this department of the State University, and his thanks to the last legislature, and the Regents of the University, for calling into existence the School of Pharmacy of the Medical Department of the University of Texas. After a few announcements of additions to the curriculum by the Dean, benediction was pronounced by Rev. Carter; and the exercises of opening the third annual session were completed.

There are matriculated to-day: First year, 45 (and about 15 or 20 more to hear from who are examined and admitted); second year, 8 (I think one or more to hear from); third year, 8.

Total, 61.

It is thought that the class will reach seventy-five or eighty by the end of the examination. A. J. S.

